# Catalytic deoxygenation of palm oil over metal phosphides supported on palm fiber waste derived activated biochar for producing green diesel fuel

**DOI:** 10.1039/d2ra03496d

**Published:** 2022-09-13

**Authors:** Napat Kaewtrakulchai, Masayoshi Fuji, Apiluck Eiad-Ua

**Affiliations:** College of Materials Innovation and Technology, King Mongkut's Institute of Technology Bangkok 10520 Thailand apiluck.ei@kmitl.ac.th +66–2–329–8625 +66–2–329–8300 ext. 3132; Kasetsart Agricultural and Agro-Industrial Product Improvement Institute, Kasetsart University Bangkok 10900 Thailand; Advanced Ceramic Center, Nagoya Institute of Technology Tajimi Gifu Japan

## Abstract

Palm oil conversion into green diesel by catalytic deoxygenation (DO) is one of the distinctive research topics in biorefinery towards a bio-circular-green economic model to reduce the greenhouse gas emissions. In this study, palm fiber waste was explored as an alternative precursor for the preparation of activated biochar as a support material. A new series of nickel phosphide (Ni–P) and iron phosphide (Fe–P) catalysts supported on palm fiber activated biochar (PFAC) was synthesized by wetness impregnation, and extensive characterization was performed by several techniques to understand the characteristics of the supported metal phosphide catalysts prior to palm oil deoxygenation for producing of green diesel (C_15_–C_18_ hydrocarbons). The PFAC support exhibited suitable physicochemical properties for catalyst preparation, such as high carbon content, and high porosity (*S*_BET_ of 1039.64 m^2^ g^−1^ with *V*_T_ of 0.572 cm^3^ g^−1^). The high porosity of the catalyst support (PFAC) significantly promotes the metal phosphide nanoparticle dispersion. The DO of palm oil was tested in a trickle bed down flow reactor under hydrogen atmosphere. The outstanding catalytic performance of supported Ni–P and Fe–P catalysts provided an impressive liquid hydrocarbon yield between 63.37 and 79.65% with the highest green diesel selectivity of 62.64%. Decarbonylation (DCO) and decarboxylation (DCO_2_) are the main pathways for the relative phosphide catalysts as presented by the high number of C_*n*−1_ atoms (C_15_ and C_17_ hydrocarbons). In addition, metal phosphide/PFAC catalysts could achieve great potential application as a promising alternative catalyst for biofuel production *via* deoxygenation for large-scale operation owing to their excellent catalytic activity, simple preparation, and utilization of sustainable resources.

## Abstract 2

Palm oil deoxygenation over palm fiber activated biochar supported metal phosphide catalysts.

## Graphical abstract

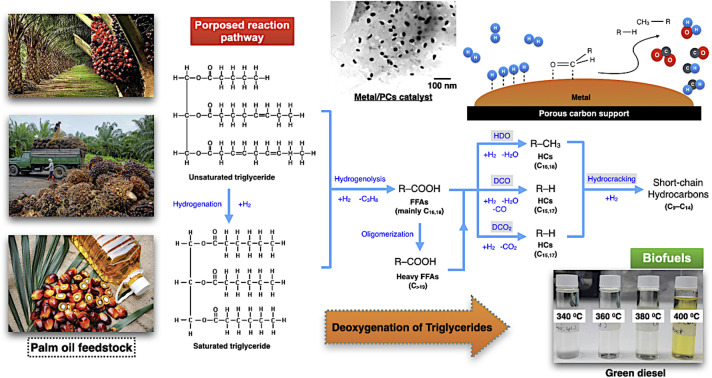

## 1. Introduction

The production of green chemicals and biofuels from renewable and environmentally sustainable resources, such as waste materials, agricultural byproducts and natural triglycerides from plants and animals, has attracted much scientific and industrial interest due to its greenness, carbon sequestration and CO_2_ emission reduction. This process could alleviate the global warming situation by replacing the use of fossil fuels.^1,2^ Presently, the consumption of petroleum in transportation and industrial sectors, particularly diesel fuel, is gradually increasing day by day owing to the fast growing economic demand and the ballooning of the world's population. The conversion of waste biomass to a green energy source has been recognized as a promising pathway to avoid using petroleum diesel in the future.^3,4^ Conventionally, biodiesel, well-developed from *trans*-esterification of natural triglycerides including plant-based oils, animal fats, and algae oils into fatty acid methyl esters (FAMEs), has become the most popular transportation biofuel. However, the drawback of biodiesel is the oxygen content, which deteriorates the relative fuel properties, such as high viscosity, low freezing point, and low thermal stability, which ultimately causes serious problems in engine systems, such as blockage of the injector and fuel filter, carbon deposition on pistons, ring sticking, cylinder head, *etc*^4,5^. To overcome these gaps, several technologies for conversion of triglycerides to efficient quality diesel-like fuel, which meet to the petroleum diesel quality need to be investigate conscientiously. Therefore, other attractive processes to convert the vegetable oils, and tallows to bio-hydrocarbons in the range of green diesel (C_15_–C_18_ paraffinic hydrocarbons) are significantly investigated. Technically, the catalytic deoxygenation essentially established for high quality of several green fuel products as though in petroleum fuels.^6,7^ Generally, catalytic deoxygenation for biofuel production is usually conducted in both hydrogen and hydrogen-free systems at 250–420 °C over moderate acidic micropore or mesopore heterogeneous catalysts under the presence of high pressure between 10–100 bar.^8^ The three parallel reactions in the deoxygenation for removal of oxygenated compounds are namely decarboxylation (DCO_2_), decarbonylation (DCO), and hydrodeoxygenation (HDO) that the contribution of each reaction pathway quite depends on types of relative reaction conditions, such as catalyst, reaction temperature, and hydrogen pressure.^9^

Currently, catalytic deoxygenation for upgrading biomass feedstocks into green fuels was run over porous materials supported noble metals (*e.g.*, palladium, platinum, and rhodium) catalysts, which is regularly employed as a commercial benchmark catalyst.^10–12^ However, the utilization of noble metals is less economically attractive than transition metal-based catalysts: nickel, cobalt, iron, molybdenum in commercialization scale. Furthermore, supported noble metal catalysts in metallic form show the poor stability in long-term catalytic run that should be urgently encountered. Over the last decade, the most important metal active phases for deoxygenation studied in several literatures are other bimetallic catalysts,^13–15^ metal sulfides,^16,17^ metal carbides,^7,18,19^ and metal phosphides,^20–22^ appears as alternative deoxygenation catalyst because of their very excellent catalytic performance, long life-time in use, and affordable price.^14,15,17,20^ Moreover, supported bimetallic catalysts showed an excellent catalytic behavior on biofuel processing, and several recent studies have done on these investigated, hence, this catalyst is still less effective for biofuels.^13–15^ Also, metal sulfide catalysts have a superior catalyst activity. However, the sulfur leaching in final hydrocarbon products is still a serious problem follows the fuel standardize. Thus, non-sulfide metal catalysts are the current research focus nowadays. Among these catalysts, phosphide metals, exhibited an excellent catalytic activity on the conversion of triglycerides into biofuels, such as bio-jet fuel, and green diesel due to its low activity for methanation that is a side reaction during deoxygenation, results in reduction of liquid hydrocarbon yield.^6,16,17^ Additionally, metal phosphide catalysts provide both metallic and acidic catalyst behaviors, corresponding to small metal positive charge leads to Lewis acid and Brønsted acid site from P–OH groups left from phosphate reduction, which is effective for oxy-compound removal.^20,21^ However, only the few endeavors have been reported in the utilization of metal phosphide catalyst for green diesel production from plant-based oil and animal fats. For example, Carmaro *et al.* demonstrated the high catalytic activity of zeolite supported nickel phosphide for producing green diesel from oleic acid.^21^ Pham *et al.* also showed the upgrading waste cooking oil into liquid hydrocarbons over activated carbon supported nickel phosphide. They revealed Ni_2_P phase presented a superior removal efficiency of oxygenated compounds, as it exhibited the great deoxygenation performance *via* decarboxylation and decarbonylation pathways.^22^ Kochaputi *et al.* reported the conversion of oleic acid to green diesel by deoxygenation using copper, nickel, and cobalt phosphide supported on silica, alumina, and zeolite.^23^ Rakmea *et al.* converted the palm oil feedstock into diesel-liked fuel over sodium mordenite supported nickel phosphide catalyst *via* hydrodeoxygenation.^24^ As mentioned above, supported metal phosphide catalysts have become the attractive candidate for green diesel production *via* deoxygenation of natural triglycerides.

In addition to deoxygenation catalysts, several recent studies have explored the utilization of high surface area materials, such as alumina, silica, zeolites, and porous carbons as metal catalyst support in the catalytic deoxygenation due to an enhancement of metal catalyst-nanoparticles dispersion and stabilization on the surface of support materials.^25^ The different support materials also display the differences in acidity and surface density of metal site, which significantly impacts the catalyst activity.^26^ To date, one of novel support materials is porous carbons developed from lignocellulosic biomass including plant-based materials and agricultural by-products. Due to this support shows some interests, such as low-cost, eco-friendly, and sustainability. Moreover, the differences in textural porosity and surface chemistry of relative porous carbon support can be facilely developed by adjusting the production parameters according to match contemplated applications. Moreover, the utilization of porous carbon as a catalyst supporter in deoxygenation inhibited a coke formation on catalyst surface during the reaction due to the neutral property of carbon materials. Also, porous carbon allows the excellent facility regarding the recovery of spent precious metal catalyst by direct burning the carbon support.^27,28^ These have been reported in recent studies as follows: Khalit and colleagues reported the usage of activated carbon from charcoal supported nickel-based catalysts in green biofuel production. The AC supported catalysts showed very effective deoxygenation activity, especially decarboxylation with hydrocarbons yields above 60% in the range of green diesel fractions (C_15_ and C_17_ hydrocarbons).^29^ Roy *et al.* studied the douglas fir derived biochar supported nickel and cobalt catalysts for hydrotreatment of carinata oil.^30^ Thangadurai also demonstrated the performance of activated carbon supported cobalt and iron oxide catalysts for the catalytic cracking of waste cooking oil to produce biohydrocarbons.^31^ Based on literature reviews, very few studies have done on investigation of utilizing waste biomass derived activated biochar supported metal phosphide catalysts in the deoxygenation of palm oil for green diesel production. This still caused a lack information on this field. To fill this knowledge gap, the approach to utilize waste biomass from palm industry as a support material for metal phosphide catalysts supplied to the production of palm oil-based green diesel offers an alternative concept in biorefinery has been explored.

Herein, we aimed to report the development of agricultural waste for producing activated biochar as a support material. The second objective of this work is to establish the alternative catalysts of activated biochar supported nickel phosphide (Ni–P) and iron phosphide (Fe–P) for producing green diesel (C_15_–C_18_ hydrocarbons) from deoxygenation of palm oil. The activated biochar support was successfully produced from palm fibers, which is one of relevant agricultural wastes collected from oil palm plantation in South-East Asia. Moreover, the development of oil palm residues to a promising catalyst for producing biofuel from oil palm feedstock effectively shows the sustainability concept of bio-refinery. The effects of experimental parameters (*i.e.* activation temperature and activating agent ratios) on the specific characteristic of palm fiber activated biochar were obviously investigated. The selected activated biochar was then applied as a support of phosphide catalysts in palm oil deoxygenation. Also, the catalytic activity of a series of synthesized phosphide catalysts was compared to the production of green diesel fuel. The selectivity to green diesel product was successfully controlled by tuning reaction conditions. However, the suitable reaction condition for producing green diesel by using these synthesized catalysts was conducted.

## 2. Experimental

### 2.1 Materials

Palm fibers was collected from consorted plantation in South of Thailand as a precursor of activated biochar processing. Nickel(ii) nitrate hexahydrate; Ni(NO_3_)_2_·6H_2_O, (98%), iron nitrate nonahydrate; Fe(NO_3_)_3_·9H_2_O (98%), potassium hydroxide (KOH), phosphoric acid (H_3_PO_4_), and hydrochloric acid (HCl) were purchased from CARLO ERBA Reagents.

Also, the palm oil, which is largely produced in Southeast Asia countries and sufficient to use for green diesel production, was supplied from local market in Bangkok, Thailand. The main composition of palm oil feedstock is described as follows: lauric acid (C12 : 0) 0.4%; myristic acid (C14 : 0) 0.8%; palmitic acid (C16 : 0) 37.8%; palmitoleic acid (C16 : 1) 0.2%; steraric acid (C18 : 0) 3.6%; oleic acid (C18 : 1) 45.8%; linoleic acid (C18 : 2) 11.1%; linolemic acid (18 : 3) 0.3%; arachidic acid (C20 : 0) 0.3%; and eicosenoic acid (C20 : 1) 0.1%. The main composition of palm oil is composed by palmitic acid and oleic acid. Thereby, palm oil appears as a potential resource for producing biofuel in diesel range hydrocarbons with huge commercial and industrial interests.

### 2.2 Synthesis of metal phosphide catalysts

Palm fiber activated biochar support (PFAC) was prepared *via* carbonization at 400 °C for 2 h under nitrogen atmosphere followed by chemical activation using potassium hydroxide (KOH) as an activating agent. The activation experiments were conducted at several conditions using activating agent ratios of 0.25 0.5, and 1.0 w/w, and different activation temperatures of 700, 750, and 800 °C under nitrogen flow of 100 mL min^−1^ for 1 h. After the activation process, the samples were completely cooled down to ambient temperature. Then, the samples were repeatedly washed with HCL, and distilled water, respectively. The washed samples were dried at 105 °C in an electrical oven for 24 h. Finally, the resulting PFAC were crushed and sieved to the uniform particle size of 0.4 mm using mesh 40 (U.S Standard Testing Sieve) for further characterization and utilization of catalyst support.

Ni–P and Fe–P catalysts were synthesized by phosphate reduction technique using an incipient wetness impregnation at 1.0 metal/phosphorus molar ratio. Among 10 wt% metal loading content was constantly used for catalyst preparation. Initially, aqueous solutions of nickel and iron precursors were prepared followed by dropwise adding of concentrated phosphoric acid. Afterwards, the PFAC was then immersed in the prepared solution and subsequently dried at 80 °C for overnight. The dried samples were pyrolyzed at 500 °C for 2 h in nitrogen flow of 100 mL min^−1^ to obtain a metal-polyphosphate species. Prior to catalyst characterization and testing in deoxygenation, the prepared samples were completely reduced under a hydrogen atmosphere to obtain the metal phosphide/PFAC catalysts.

### 2.3 Characterization of PF activated biochar and catalysts

The chemical composition of raw PF and prepared PFACs including moisture, volatile matter, fixed carbon, and ash was determined by proximate analysis.^32^ The %Moisture, was calculated by drying process for 24 h at 105 °C follows the American Society for Testing Materials, ASTM D2867-99 method (ASTM, 2014).^33^ %Volatile matter was measured by carbonization according to an ASTM D5832-98 (ASTM, 2008).^33^ %Ash was analyzed by direct combustion follows ASTM D2866-94 (ASTM, 2011).^33,34^ In addition, a solid carbon remaining in the sample referred to as %Fixed carbon, is reported by subtracting moisture, volatile matter, and ash contents from 100%.^32^ Also, the elemental compositions (carbon: C, hydrogen: H, and nitrogen: N contents) of palm fibers and PF activated biochar were measured by the CHN elemental analyzer (Leco truespec CHN-628).

The porosity of PFACs and synthesized catalysts were measured by nitrogen physisorption at −196 °C on the Quantachrome Autosorp iQ-MP-XR. The samples were pretreated at 300 °C for 3 h before the analysis begins. The determination of surface areas was computed by BET (Brunauer–Emmett–Teller) model. The pore size distribution was measured by the DFT (density functional theory) method, and the total pore volume (*V*_T_) was calculated by a condensation of liquid nitrogen at the relative pressure of 0.99 using the Quantachrome ASiQwin 2.0 software. In addition, the micropore volume (*V*_mi_) was determined by the t-plot model. The mesopore volume (*V*_me_) was calculated by subtraction of *V*_mi_ from *V*_T_.^35^

The species of metal phosphide catalysts were characterized on X-ray diffractometer (SmartLab, Rigaku, Japan) operated at 40 kV and 40 mA using Cu-Kα radiation (*λ* = 1.5406 Å). The 2*θ*-scanning range was from 15 to 80°, and the scanning rate was 0.5° s^−1^. The crystallite size of metal phosphide catalysts was measured following the Debye–Scherrer equation.

Surface morphology of PFAC support and synthesized catalysts were observed on a scanning electron microscope, SEM (Zeiss EVO50, Germany) operated at 20 kV, FE-SEM (Hitachi SU8030, Japan), and a transmission electron microscope, TEM (JEOL JEM-2100, Japan) operated at 200 keV, respectively.

The acidity of metal phosphide catalysts was carried out by using ammonia-temperature programmed desorption (NH_3_–TPD) technique (5% NH_3_/He) in a Quantachrome Chemisorption Analyzer ChemStar (TPX Series), equipped with a thermal conductivity detector.

Surface functional characteristics of PFAC support was characterized by using Fourier transform infrared spectroscopy (FTIR). The infrared adsorption spectrum is the wavenumber ranges from 4000–400 cm^−1^. The samples were put into infrared platform and impressed directly before to start characterization in transmittance mode on FTIR spectrometer, PerkinElmer UATR Two.

The study of surface chemical compounds of PFAC support, and metal phosphide catalysts were conducted by using X-ray photoelectron spectroscopy (XPS), Kratos AXIS supra (XPS) surface analytical with Al Kα radiation as the excitation source. The samples were situated on the carbon tape placed on the sample stub and substituted to high vacuum system before measurement. All binding energy spectra were deconvoluted by XPS Kratos ESCApe data processing software to fit the desired spectra, such as C 1s, O 1s (for carbon support), Ni 2p, Fe 3p (for studied catalysts), respectively.

### 2.4 Palm oil deoxygenation

The deoxygenation performance of Ni–P and Fe–P catalysts on green diesel production was investigated in the continuous down-flow reactor under different reaction conditions. 8 mL of Ni–P and Fe–P catalysts were used in the DO of palm oil follows the feedstock flow rate at liquid hourly space velocity (LHSV) of 1 h^−1^. The synthesized catalyst was packed into the middle of stainless-steel reactor tube, and then *in situ* reduced immediately under H_2_ flow of 50 mL min^−1^ at 650 °C for 3 h by using a ramping rate of 5 °C min^−1^ to ensure that the metal phosphide phase was achieved for the reaction. After the catalyst reduction, the system was cooled down to the desired reaction temperatures of 340, 360, 380, and 400 °C, and then pressurized with hydrogen of 50 bar using a back-pressure regulator under the controlled H_2_ flow of 100 mL min^−1^. The reaction test was evaluated after the start up time of 4 h. This is for reaching steady state of catalytic reaction. The fresh catalyst was used in all experiments to assure the catalyst performance because the catalyst deactivation can be occurred after each experimental run.^36^ In product analysis, the liquid product collection was periodically conducted at every 2 h intervals for analysis to obtain the average value. The 50 mg of liquid product compositions after separation of water phase was diluted with 1 ml of hexane, and 1 μL of prepared sample was characterized by offline gas chromatography equipped with a mass spectrometer detector (GCMS-QP2020, Shimadzu, Japan) using capillary column (DB-1HT, 30 m × 0.32 mm x 0.1 μm) and a flame ionization detector (FID). The temperature of injection port and detector was set at 370 °C. The analyzed mass spectra of C_8_–C_20_ hydrocarbons were matched with the National Institute of Standard and Testing (NIST) library. The conversion of palm oil, liquid hydrocarbon yield (HCs, C_9_–C_24_), and green diesel (C_15_–C_18_) selectivity were calculated by using the following equations (eqn. (1), (2) and (3)), respectively.1
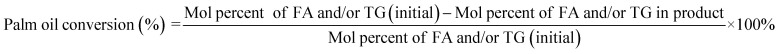
2
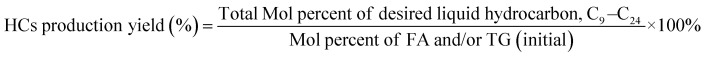
3
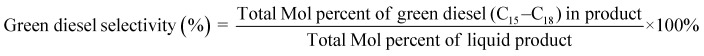


## 3. Results and discussion

### 3.1 Characterization of activated biochar support

#### 3.1.1. Proximate and ultimate analyzes

Chemical contents and elemental compositions of raw PF, PF biochar, and PF derived activated biochar were characterized by using proximate and ultimate analysis, as listed in Table 1. The results demonstrated in dried-basis condition. The proximate analysis of raw PF feedstock has 72.24% volatiles, 6.13% ash, and 21.63% fixed carbon, while the ultimate analysis shows that the elemental compositions were 46.73 ± 1.35% C, 2.27 ± 0.43% H, 1.36 ± 0.12% N, and 49.64 ± 3.32% O, respectively. According to proximate and ultimate analyzes, raw PF can be used as a high potential resource for producing carbon material due to its high carbon and fixed carbon contents. This composition is significantly affected on the high production yield of resulting carbon material.^37,38^

**Table 1:** Ultimate and proximate analyzes of palm fibers and PFAC

Samples	Proximate analysis (dried basis, %)			Ultimate analysis (dried basis, %)			
	VM	FC^a^	A	C	H	N	O^a^
PF feedstock	72.24 ± 2.47	21.63 ± 0.52	6.13 ± 0.18	46.73 ± 1.35	2.27 ± 0.43	1.36 ± 0.12	49.64 ± 3.32
PF biochar	29.26 ± 0.45	62.21 ± 3.96	8.53 ± 0.53	66.49 ± 4.12	1.64 ± 0.25	1.12 ± 0.06	30.75 ± 1.38
PFAC700-0.25	20.43 ± 2.19	68.38 ± 5.74	9.19 ± 1.31	70.75 ± 5.04	1.04 ± 0.17	1.06 ± 0.02	27.15 ± 1.24
PFAC700-0.5	20.62 ± 1.83	71.14 ± 7.12	8.24 ± 0.58	72.44 ± 2.35	1.02 ± 0.26	1.01 ± 0.07	25.53 ± 1.13
PFAC700-1	21.26 ± 1.48	69.49 ± 4.65	9.25 ± 0.75	72.16 ± 1.83	0.98 ± 0.12	0.95 ± 0.03	25.91 ± 0.95
PFAC750-0.25	17.72 ± 0.63	74.37 ± 2.24	7.91 ± 1.03	75.19 ± 2.14	0.97 ± 0.11	1.06 ± 0.08	22.78 ± 1.21
PFAC750-0.5	18.89 ± 1.25	72.49 ± 1.28	8.62 ± 0.70	74.37 ± 1.21	0.92 ± 0.09	0.99 ± 0.05	23.72 ± 1.07
PFAC750-1	17.04 ± 2.81	73.61 ± 2.49	9.35 ± 1.29	77.62 ± 1.09	0.93 ± 0.14	1.01 ± 0.09	20.44 ± 1.15
PFAC800-0.25	12.53 ± 2.58	79.71 ± 3.85	7.76 ± 1.32	79.94 ± 4.73	0.83 ± 0.16	0.96 ± 0.03	18.27 ± 0.98
PFAC800-0.5	15.86 ± 0.93	76.64 ± 5.12	7.50 ± 0.97	78.26 ± 2.07	0.89 ± 0.11	0.97 ± 0.06	19.89 ± 1.19
PFAC800-1	13.02 ± 1.16	78.64 ± 6.27	8.34 ± 0.85	80.31 ± 1.42	0.92 ± 0.08	0.93 ± 0.04	17.97 ± 1.06

a Calculated by difference, M: moisture, VM: volatile matter, FC: fixed carbon, A: ash; (means ± SD; *n* = 3).

Nonetheless, the carbonization was firstly carried out at 400 °C for 2 h for pretreatment of a raw PF to develop the fixed carbon percent by releasing volatile and oxygenated compounds to produce a solid carbon before activation process. The value of carbon percentage and fixed carbon content significantly increased, when compared to proximate and ultimate analyzes of PF due to the thermal decomposition of lignocellulosic compounds that occurred a devolatilization. Besides, the percentage of oxygen was lower than that of PF feedstock suggesting that moisture and volatile matters are removed.^39^ Moreover, the ultimate and proximate analyzes of activation samples (PFAC) with different conditions of activation temperature and dosage of KOH activating agent are also demonstrated in Table 1.

According to proximate analysis, PFAC samples have a fixed carbon content between 68.38 and 79.71%. It was found that the fixed carbon content essentially gets raised by increasing activation temperature (700–850 °C), while the activating agent ratio slightly impacted on the percentage of fixed carbon. This is due to the high temperature in PFAC manufacturing is significantly released the volatile matters *via* devolatilization, and this also partially converted the oxygenated compounds into solid carbon *via* graphitization, simultaneously.^39,40^ On the other hand, volatile content also reduced with increasing activation temperature in the range from 12.53–21.26%. This finding consisted with the reducing oxygen content in ultimate analyzes of PFAC, which results in a higher hydrophobicity. One of crucial parameters in the proximate analysis of carbon material derived from biomass is ash content since the ash might cause an influence on a significant role in specific applications such as hydrophilicity and some catalytic effects.^33,39,41^ The prepared activated biochar derived from palm fiber contains low ash content (7.50–9.35%).

The ultimate analyzes showed that PFACs was approximately 70.75–80.31% C, which is quite high when compared to a commercial activated carbon (85–90% C).^42^ Noticeably, the percentage of carbon in activated carbon is always seemingly proportional with its surface area value, which the majority property of activated carbon sample is a porosity and surface area since the pore development is mainly occurred by partial oxidation of carbon atoms during activation process, while the high content of inorganic substances (ash composition) may suppress the porosity enhancement results in reducing specific surface area and porosity, directly.^33,41^ Hence, the high carbon content of PFACs may be desirable for the excellent pore formation. The highest surface area approximately 1039.64 m^2^ g^−1^ was obtained from PFAC750–0.25 with %C of 75.19%. Table 1 also shows the N and H contents of PFAC samples. The small amount of N was found nearly less than 1% (0.93–1.06%). This can be left from the nitrogen content in plant structure and partially chemisorbed during activation stage.^41^ Hydrogen content was also observed between 0.83 and 1.04%, which is normally a chemical H bonding with carbon atom in carbon structure.^43^ Additionally, PFAC samples have O content 17.97–27.15%. It was gradually decreased from 49.64% O in a raw PF. This is due to the releasing of volatiles and oxygenated compounds in the plant-based biomass during high-temperature processing, which the results are in good agreement with the study of Mahamad *et al.*^44^ and Zubrik *et al.*^38^ According to the finding results, it was found suggested that both chemical contents and elemental compositions of PFACs were strongly influenced by the high temperature during activation process. These analyzes have a good agreement with several literatures, which reported the similar observations that increasing activation temperature significantly obtained the product with higher carbon percentage and fixed carbon content due to the influence of thermal decomposition reactions during activation occurred simultaneously. In addition, the enough activating agent may decrease in fixed carbon content.^37,38,43,45^

#### 3.1.2. Characterization studies on porous structure of PF activated biochar (PFAC): effect of activation conditions

**Fig. 1:**
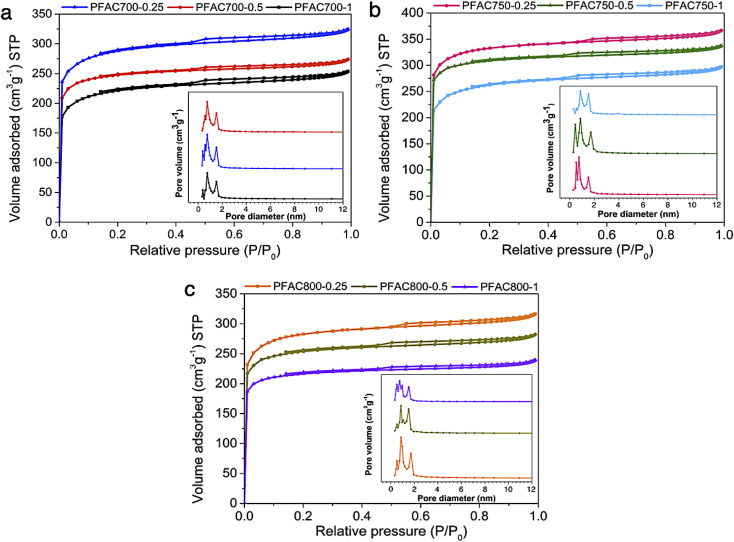
N_2_ adsorption/desorption isotherms and pore size distribution of PFAC samples obtained at (a) 700 °C, (b) 750 °C, and (c) 800 °C with different KOH ratio.

N_2_ sorption isotherms and pore size distributions of all PFAC samples are displayed in Fig. 1. Textural pore characteristics including BET surface area, total pore volume, and average pore size are listed in Table 2. The adsorption–desorption isotherm of PFACs obtained from different activation temperatures of 700, 750, and 800 °C with different KOH ratios of 0.25, 0.5, 1 are shown in Fig. 1a–c, respectively. The isotherms of all samples are the Type I of sorption isotherms follows the International Union of Pure and Applied Chemistry (IUPAC) classification corresponds to microporous materials.^45^ However, a narrow hysteresis loop at the relative pressure between 0.45 and 0.99, according to the mesoporous materials, are also observed indicating that the obtained carbon samples have mesopores in their carbon matrix. Also, the findings are ensured with the pore size distributions, which shows 2 main distributions: the one is mainly in the narrow range of micropores (<2 nm), and another one is some narrow mesopores sizes approximately around 2 nm. It was suggested that PFACs samples have a micro/meso porous structure. This characteristic is specifically observed form carbon materials derived from lignocellulosic biomass.^46,47^

**Table 2:** Textural pore characteristics of PF activated biochar (PFAC)

Samples	*S*_BET_ (m^2^ g^−1^)	*V*_total_ (cm^3^ g^−1^)	*V*_micro_ (%)	*V*_meso_ (%)	*D*_AVG_ (nm)
PF biochar	178.35	0.104	35.52	64.48	3.13
PFAC700-0.25	893.85	0.505	57.36	42.64	2.55
PFAC700-0.5	717.26	0.398	61.09	38.91	1.98
PFAC700-1	629.39	0.346	58.23	41.77	2.06
PFAC750–0.25^a^	1039.64	0.572	71.66	28.34	1.82
PFAC750-0.5	937.31	0.486	60.31	39.69	1.99
PFAC750-1	830.25	0.441	61.75	38.25	1.97
PFAC800-0.25	975.21	0.517	68.63	31.37	1.89
PFAC800-0.5	813.98	0.438	56.68	43.32	2.04
PFAC800-1	705.33	0.392	63.69	36.31	1.98

a The highest porosity of PFAC that was applied as a metal catalyst support in palm oil deoxygenation.

The typical PFAC samples have total pore volume (*V*_total_) of 0.346–0.572 cm^3^ g^−1−1^. However, PFACs also have a micropore volume of 56.68–71.66%, and mesopore volume (*V*_meso_) of 28.34–42.64%, respectively. The surface area (*S*_BET_) of all PFACs is higher than the PF biochar without activation. Technically, the activation process significantly develops the surface area of carbon materials. An increase in surface area and porosity enhancement depends on the activation parameters, such as activation temperature, dosage of activating agents, and types of activating agents.^33,39,48^ In this study, KOH was used as an activating agent, which is one of effective chemicals for well development of superior surface area and porosity in the porous carbon production.^48,49^

According to activation temperature, the effect of activation temperature (700, 750, and 800 °C) on *S*_BET_ of PFACs are shown in Table 3. The pore characteristics of PFACs are significantly influenced. The increasing surface area and porosity are generally correlated with the increasing activation temperature to reach the highest porosity and *S*_BET._ However, the porosity and surface area might be decreased at the exceed activation temperature stage due to the excessive energy for activation reaction leads to the pore ablation and micropore sintering.^48^ Also, the KOH dosage is the crucial factor to maximize the *S*_BET_ of PFACs. Furthermore, increase in KOH until excess exhibited the highest *S*_BET_ and even their porosity. More excess activating agent gradually decreased in *S*_BET_ because the optimum porous structures of PFACs, which completely formed at the excess KOH activation stage, was broken leading to the reduction of *S*_BET_ and porosity.^50^ The PFAC750–0.25 has a superior pore structure than the others with the highest *S*_BET_ of 1039.64 m^2^ g^−1^, and *V*_total_ of 0.572 cm^3^ g^−1^, respectively. Nonetheless, the activation using the condition of KOH ratio of 0.125 at 750 °C was also tested to assure that the condition of 750 °C using 0.25 KOH ratio is the real optimum condition to obtain PFAC with highest porosity. The results showed that PFAC750–0.125 has a *S*_BET_ of 831.45 m^2^ g^−1^, and *V*_total_ of 0.446 cm^3^ g^−1^. In addition, the PFAC750–0.25 exhibited the special properties, such as high surface area, and porosity, high carbon content for its feasibility to be utilized as a support material. Therefore, the PFAC750–0.25 was selected as a supporter of metal phosphides on the producing green diesel *via* palm oil deoxygenation.

**Table 3:** Functional groups of lignocellulosic materials

Wavenumber (cm^−1^)	Functional group	Description
3680–3000	O–H stretching	Hydroxyl or carboxyl groups, alcohol from cellulose or phenols from lignin
2925	C–H stretching	Aliphatic
1700	C=O stretching	Carbonyl, ester or carboxyl from cellulose and lignin
1600	C=C stretching	Aromatic skeletal present in lignin
1290–950	C–O stretching	Ester from hemicellulose
860–724	C–H bending	Aromatic
<500	Metal-O stretching	Inorganic compound (ash)

#### 3.1.3. Surface morphology of PFAC

**Fig. 2:**
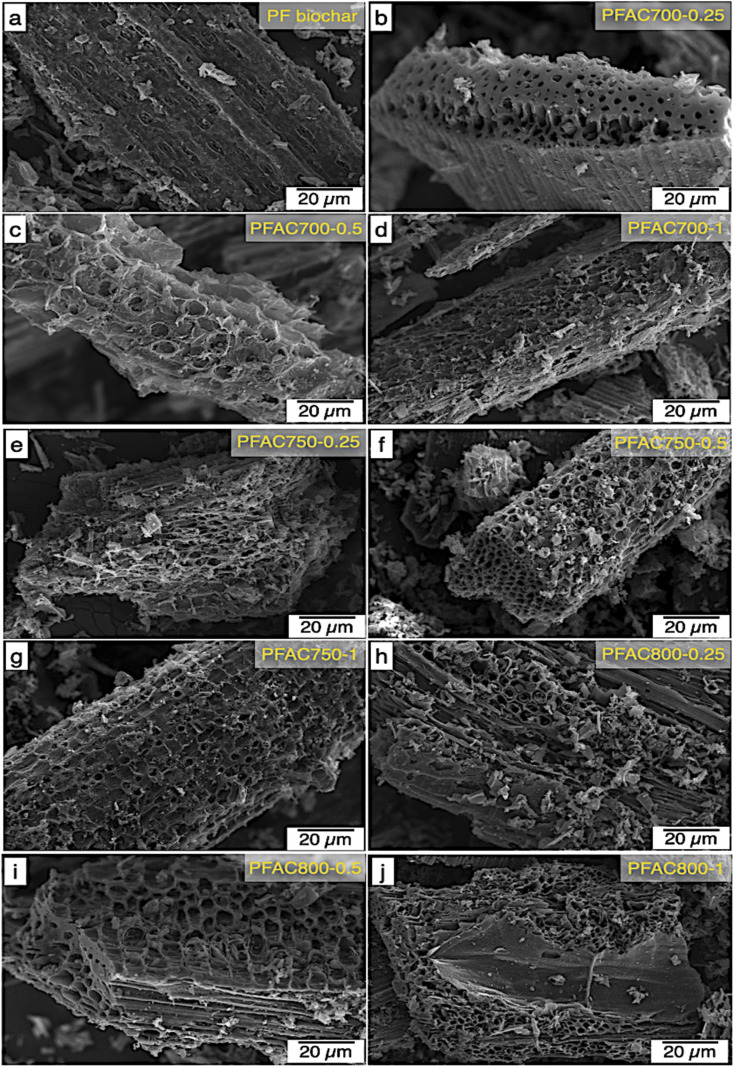
SEM micrographs of PF biochar and PFAC.

The surface morphology of PF biochar obtained by carbonization as well as the PFACs obtained from different processing conditions was observed by using Scanning Electron Microscope (SEM), as presented in Fig. 2. In comparison to the biochar derived from pretreatment step, the activation temperature and dosage of KOH were analyzed on the surface morphology of PFACs. The SEM micrograph of PF biochar appeared the dense surface with minimum visible pore cavities (Fig. 2a), while appearance of porous structures was found on the PFAC carbon matrix after the activation was completely conducted. This suggested the partial oxidation upon KOH activation between carbon atom and KOH molecules occurred at high temperature process resulted in new pores formation,^37^ as illustrated in Fig. 2b–j.

As shown in the SEM micrographs, the observation revealed that the increase of activation temperature and KOH dosage significantly caused a changing with the appearance of PFAC surface morphology in terms of enhance the extensive external pore cavities, which are the entry way into internal pores (mesopores an micropores). However, the observation on the PFACs surface morphology is correlated with their porous structure analyzes. This suggests that the PFACs with high surface area can be observed the appearance of various pores on its external surface, as compared with Table 2.

#### 3.1.4. Crystal structure and surface chemistry of PFAC

**Fig. 3:**
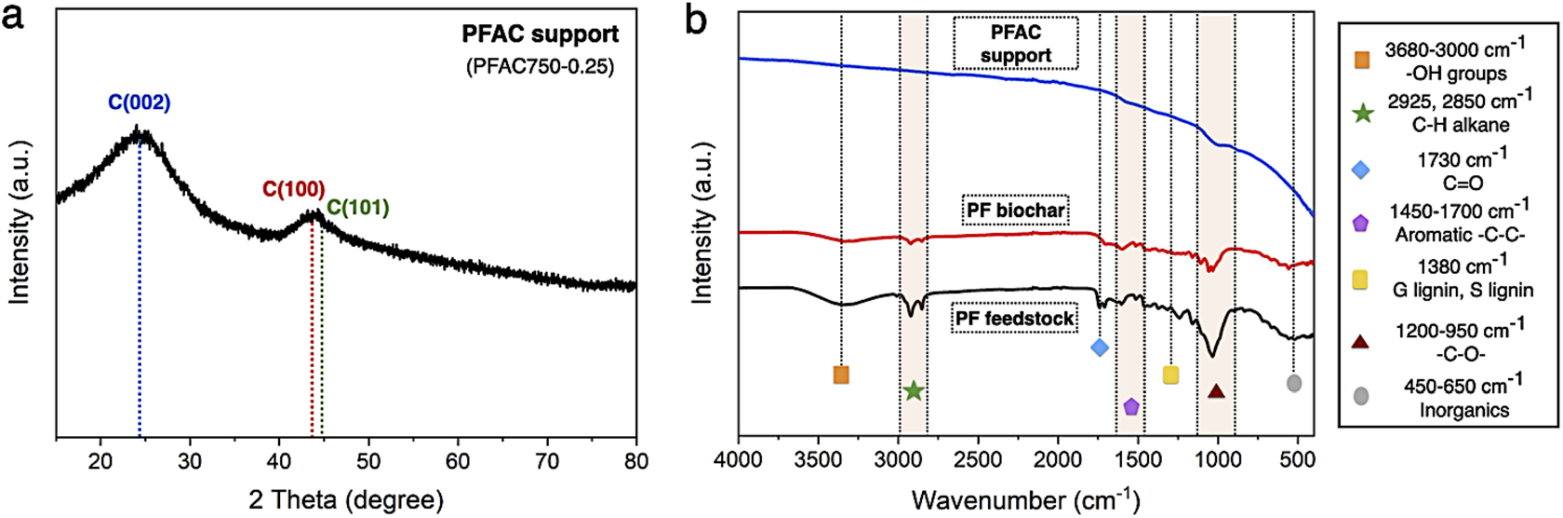
Characterization of PFAC support (a) XRD pattern, and (b) surface functional group analyzes.

**Fig. 4:**
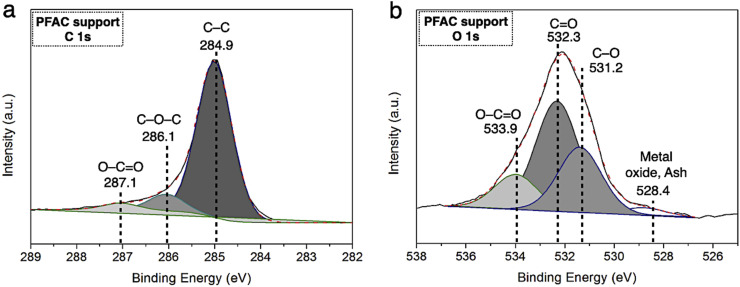
XPS spectra of PFAC support (a) C 1s spectra, and (b) O 1s spectra.

The characterization on crystallinity structure and surface chemistry of PFAC support (PFAC750–0.25) was evaluated by using powder X-ray diffractometer, XRD, Fourier transform infrared spectroscopy, FTIR and X-ray photoelectron spectroscopy, XPS techniques, as displayed in Fig. 3 and 4, respectively. The XRD pattern of PFAC support was observed in the 2*θ* range of 15–80°, as shown Fig. 3a. The domination of amorphous structure from PFAC support was observed with a broad and smooth pattern. The XRD pattern of PFAC support is good agreement with the standard file no. JCPDS 41-1487.^51^ The broad peak around 19–26° represented the intensity of the (002) diffraction facet for amorphous carbon. Also, the crystal planes of (100), and (101) ascribed to graphitic carbon layer are observed at 2*θ* of 43° and 44.8°.^51^ However, the XRD patterns of PFAC support compared to the other synthesized catalysts revealed the crystal structure changes from PFAC to typical catalysts, which showed the appearance of metal phosphide phases such as Ni–P, and Fe–P crystals on the PFAC network, as demonstrated in Fig. 7b.

In Fig. 3b, the FTIR spectra of PF feedstock, PF biochar, and PFAC support were determined by the infrared absorption spectra using direct transmittance in the wavenumber ranges from 4000–400 cm^−1^, where the surface functionalities of lignocellulose materials are explained in Table 2. FTIR analyzes shows the appearance of several chemical functions. The polycyclic aromatics from lignin in plant-based biomass are including –OH stretching of hydroxyl and carbonyl groups at 3680–3000 cm^−1^, and aromatic –C=C– rings between 1600 and 1512 cm^−1^.^51^ Moreover, aliphatic C–H groups of polysaccharides in cellulose and hemicellulose was at the vibration bands between 2925 and 2850 cm^−1^.^52^ However, the stretching bands of lignin composition is also observed at 1380–1240 cm^−1^ and 1460 cm^−1^. In addition, the band of C=O, and –C–O– vibrations correspond to cellulose and hemicellulose structure was at 1550–1730 cm^−1^, and 1200 and 950 cm^−1^, respectively. Some inorganic molecules or metal compounds, which known as ash could be necessary observed at 500 cm^−1^ of the intense band.^51,52^ Finding results exhibited the similar FTIR spectra frameworks of PF feedstock, PF biochar, and PFAC support, as displayed in Fig. 3b. In FTIR spectra of PFAC support, the symmetrical stretching of organic functional groups, which greatly disappeared from the observed spectra because the surface functional groups were successfully decomposed. The decrease in the observed FTIR intensity was occurred by thermal decomposition of lignocellulosic components at high temperature during PFAC manufacturing.^53^ Moreover, the FTIR spectra of PMFC cannot observed on the relative intense bands below 500 cm^−1^, indicating that PFAC support consisted of a low ash content.

The resolutions of C 1s and O 1s spectrum of PFAC support (PFAC750-0.25), which are obtained by XPS analysis, are displayed in Fig. 4. In Fig. 4a, C 1s spectrum can be revolved into three component peaks which represented the peak intensity of graphitic carbon (–C=C–, 284.75 eV), the group of carbon in alcohol and/or ether linkage (C–O–C, 286.23 eV) and carbon in carbonyl group (O–C=O, 288.79 eV), respectively. The high intensity peaks centered at 284.75 eV, suggesting that PFAC support has a high proportion of graphitic carbon on its surface.^39,45^ Also, it can be indicated that PFAC support composed of high C content. This could be larger content than PF feedstock and PF biochar. These results are consistence with the ultimate analyzes (Table 1). Besides, O 1s spectrum (Fig. 4b) exhibited the four relevant spectra representing C–O in phenols and ethers groups at 531.2 eV. Also, the organic C=O in carboxylic acid and/or ester group was centered 532.3 eV.^45^ The O–C=O of organic molecule in carbon support was demonstrated at 534.0 eV, and metal oxide or inorganic molecules ascribes to ash content in carbon support was at 528.4 eV.^45,54^ It was found that PFAC support (PFAC750-0.25) has a low shoulder peak, as seen in O 1s spectrum. This can be implied that PFAC support contains a low ash content. This result is consisted with the information of chemical composition from proximate analysis, as shown in Table 1.

### 3.2 Catalyst characterization

**Fig. 5:**
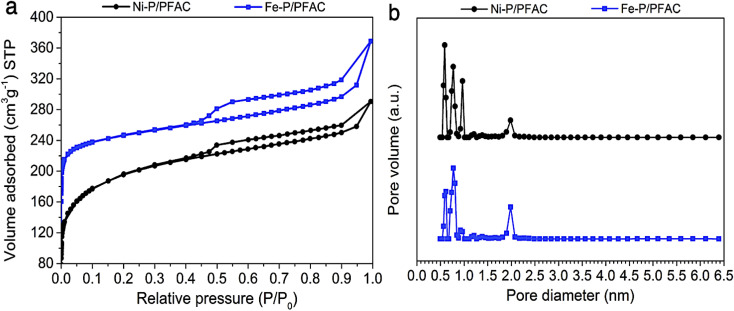
Porosity analyzes of studied catalysts (a) N_2_ adsorption–desorption isotherms, and (b) Pore size distribution curves.

Fig. 5a shows the N_2_ adsorption–desorption isotherm of reduced metal phosphide catalysts before testing on palm oil deoxygenation. The sorption isotherms of both Ni–P/PFAC and Fe–P/PFAC catalysts obviously has a mixing of Type I and Type IV characteristics according to the IUPAC classification, implying that the obtained catalysts show a combination of microporous and mesoporous structures.^54,55^ The adsorbed volumes on supported Fe–P and Ni–P are lower than the bare PFAC support due to the instead of metal nanoparticles on the carbon surface. This could be decrease in the pore volume (seen in Tables 3 and 4). Nonetheless, the reduction of catalysts before characterization and reaction testing may decrease in total pore volume because of the pore collapse by thermal decomposition during high temperature operation.^56,57^ The surface area of Ni–P/PFAC and Fe–P/PFAC catalysts are 718.65 m^2^ g^−1^ and 850.92 m^2^ g^−1^, respectively (Table 4). Also, the pore size distributions of corresponding catalysts are displayed in Fig. 5b. The size distributions are in the range of 0.5–2.5 nm, which the main pore size distribution was in micropores range (<2 nm), and the narrow mesopore size distribution occurred at 1.9–2.5 nm. However, Table 3 also presented that a higher proportion of micropore volume than mesopore were observed from the obtained catalysts. The high porosity and surface area of synthesized catalysts can be implied that very well dispersion of metal nanoparticles was achieved on the PFAC surface. This indicates the essential characteristics of metal catalyst for catalytic deoxygenation resulting in excellent catalyst activity.^58^

**Table 4:** Physical and chemical properties of PFAC supported Ni–P and Fe–P catalysts

Catalysts	*S*_BET_ (m^2^ g^−1^)	*V*_T_ (cm^3^ g^−1^)	*V*_mic_ (%)	*V*_mes_ (%)	Crystallite size (nm)	NH_3_ uptake (μmol g^−1^)
Ni-P/PFAC	718.65	0.437	68.29	31.71	10.58	119.54
Fe-P/PFAC	850.92	0.511	61.45	38.55	6.13	144.72

**Fig. 6:**
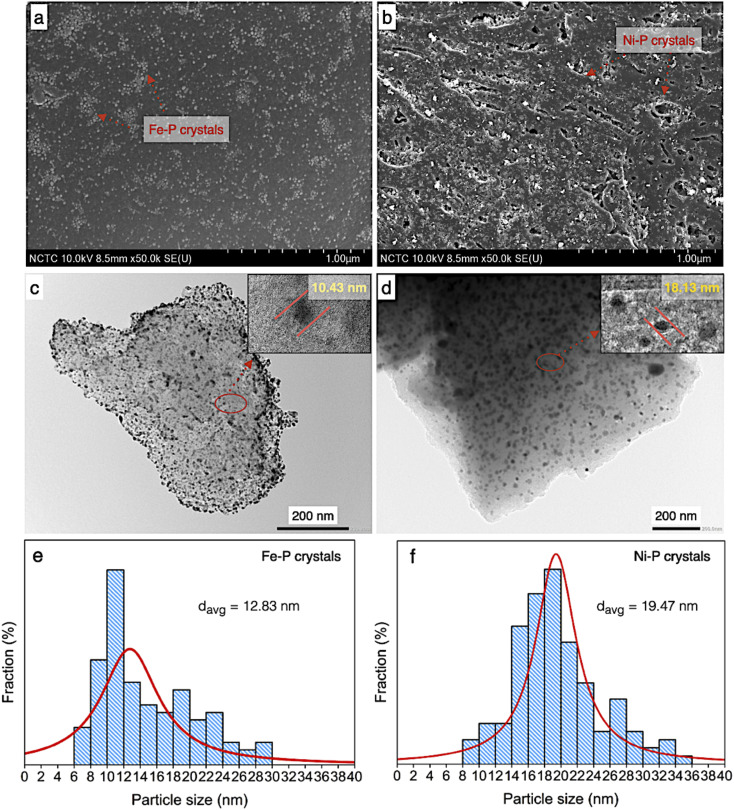
Physical morphology of PFAC supported Ni–P and Fe–P catalysts; FESEM images of (a) Fe–P/PFAC, (b) Ni–P/PFAC, TEM images of (c) Fe–P/PFAC and, (d) Ni–P/PFAC, and particle size distribution (e and f).

According to morphology observation of catalysts, the SEM, TEM, and particle size distribution of both supported Ni–P and Fe–P are displayed in Fig. 6. In the same implication on SEM micrograph, the clusters of spherical shape of both Ni–P and Fe–P nanoparticles were obtained with well distribution on carbon support, as seen in Fig. 6a and b, respectively. This result implies that the active sites is significantly increased resulting in the improvement of catalyst performance. Additionally, the observation on dark spots ascribes to Ni–P and Fe–P is also shown in TEM image (Fig. 6c and d). This indicated that Ni–P and Fe–P nanoparticles are well dispersed on both the surface and inside of PFAC support. However, the low scattering contrast of the dark spots referred to the corresponding Ni–P and Fe–P were observed. It was implied that metal phosphides nanoparticles probably locate both inside and outside pores of PFAC supports. The porosity and surface area of PFAC support were gradually decreased after the deposition of metal phosphide, indicating that the metal phosphide nanoparticles might cause the pore filling.^59,60^

According to TEM micrographs, the Fe–P nanoparticles seemingly better disperse on the PFAC in comparison to Ni–P. The average particles sizes and particle size distribution (Fig. 6e and f) are also correlates with TEM results. Fe–P and Ni–P nanoparticles has an average size of 12.83 and 19.47 nm, respectively. Moreover, the particle size distribution of Fe–P was observed between 6 and 30 nm, which the main distribution is at 6–14 nm. In contrast, the particle size distribution of Ni–P was found with shifting on the right axis within 8–36 nm. The distribution of Ni–P size was mainly between 14 and 24 nm. Besides, the crystallite sizes from XRD analysis (Table 4), which calculated by using Debye–Scherrer equation, showed that Fe–P and Ni–P nanoparticles is approximately 6.13 and 10.58 nm, respectively. Remarkably, well dispersion of metal phosphide crystals results in high active surface area of obtained catalysts is a promising characteristic for enhancement of catalyst activity. This can be developed from porous carbon materials derive from agricultural waste.^61^

**Fig. 7:**
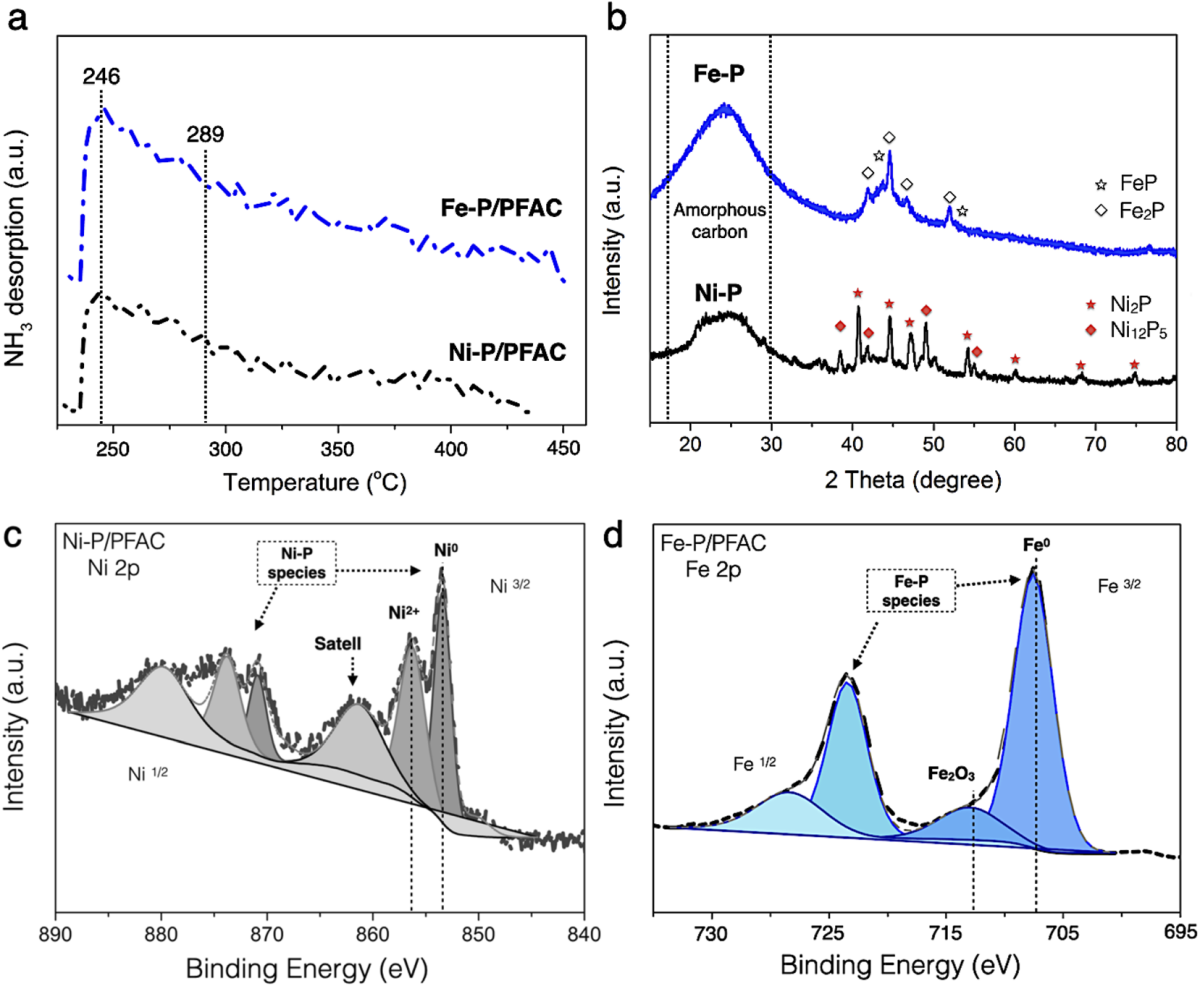
Characterization on of PFAC supported Ni–P and Fe–P catalysts (a) temperature programed desorption (NH_3_-TPD), (b) XRD pattern of studied catalysts, (c) XPS spectra of Ni–P/PFAC and, (d) XPS spectra of Fe–P/PFAC.

The acidity of obtained catalysts after reduction process is shown in the NH_3_-TPD profiles, as seen in Fig. 7a. The amount of desorbed NH_3_ is reported in Table 4. The profiles of desorbed NH_3_ of corresponding catalysts have a dominant desorbed peak at approximately 246 °C that correlated with the characteristic of NH_3_-desorption at the low temperature between 100 and 250 °C corresponds to weak acidity, the Brønsted acid site, particularly conducted from the surface of M − OH and P–OH groups in unreduced or phosphates species of catalysts.^62^ This might occur during preparation for the catalyst characterizations. However, the strong shoulder peak also observed at the temperature around 289 °C corresponds to moderate and strong acid sites (the Lewis acid), which is specifically attributed to the characteristic of some M^*δ*^+^^ charges of Ni–P and Fe–P nanoparticles. This characteristic could be due to a greater electron mobility in the metal phosphide structure.^63^ Consuelo Alvarez-Galval *et al.*^64^ also reported that the intense NH_3_ desorption peak of reduced metal phosphides was found in the ranges of moderate and strong acid sites (>250 °C). As the observation, the NH_3_ desorption peak of supported Fe–P catalyst showed a strong intense and broader shoulder peaks at high temperature than Ni–P/PFAC catalyst, which also showed the larger acid sites and stronger acidic were obtained from Fe–P/PFAC. Also, the total desorbed NH_3_ amount of Fe–P/PFAC and Ni–P/PFAC catalysts were obtained approximately 144.72 and 119.54 μmol g^−1^, respectively. This consequence is due to the better dispersion and smaller size of Fe–P crystals resulted in the increasing NH_3_ adsorption on Fe–P surface.^64,65^ Moreover, strong acidity catalysts significantly exhibited an excellent activity in deoxygenation of oxygenated compounds since the high acidity is favored to the cleavage of C–C and C–O bonds. In addition, the hydrocracking reaction for efficient production of light hydrocarbons is also represented in the similar contribution.^66,67^

Fig. 7b shows the XRD diffraction pattern of obtained catalysts at the 2*θ* angle 15–80°. The broad peak of diffraction between 15–30° could be ascribed to amorphous carbon of PFAC support, as illustrated in Fig. 3a. Additionally, average crystallite size of metal phosphide was calculated by the Scherrer equation using XRD peak intensity of relative catalyst, as listed in Table 4. The average crystallite size of Fe–P and Ni–P was about 6.13 and 10.58 nm, respectively. The average domain sizes of corresponding catalysts were correlated to the particle sizes from the TEM analysis (Fig. 6). The XRD patterns of both supported metal phosphide catalysts were a sharp peak with high intensity suggesting that the crystalline phase was obtained. The supported Ni–P catalyst was observed the intense peak at 2*θ* angle of 35–75°. The intense peaks at 2*θ* of 40.7, 44.6, 47.4, 54.2, 59.9, 69.7, and 74.6° were mainly Ni_2_P crystal (PDF 65-1989), also, low intensity peaks at 2*θ* of 38.3, 42.2, 49.9, and 55.0° corresponds to Ni_12_P_5_ (PDF 22-1190). The transformation of Ni_2_P to Ni_12_P_5_ could be occurred during pyrolysis and reduction process in the catalyst synthesis.^68^ According to supported Fe–P catalyst, the XRD pattern was an overlapped diffraction lines between 2*θ* angle of 40–55°, which ascribed to the Fe_2_P (PDF 85-1725) (2*θ* = 41.8, 44.5, 46.9, and 52.1°), and FeP (PDF 81-1173) (2*θ* = 43.9 and 53.7°) phases.^64^ This also indicates that the Fe_2_P could be transform to FeP during catalyst preparation. The high temperature and long resident time in the preparation might influence on the ratio of metal/P by partial releasing phosphorus as a volatile species such as phosphine (PH_3_) resulted in the phase transformation.^69^

The surface chemistry of corresponding catalysts in a reduced form were observed by XPS, as displayed in Fig. 7c and d. The XPS spectra were reported at binding energies of Ni 2p_3/2_ and Fe 2p_3/2_ core levels, respectively. The binding energy of Ni 2p was separated in two main contributions at 853.7 eV, attributed to metallic nickel (Ni^*δ*^+^^ species) in metal phosphide, and approximately 856.4 eV, which was accompanied by a broad satellite peak is assigned to Ni^2+^ in unreduced PO^3−^/PO^4−^, respectively.^68–70^ Noted that, the Ni^2+^ in unreduced probably formed by re-oxidation with air during the catalyst preparation for XPS analysis.^70^ According to supported Fe–P catalyst, the Fe 2p_3/2_ spectra are also shown in two main contributions. The sharp peak at binding energy of 707 eV is corresponded to the binding energy of metallic iron (Fe^*δ*^+^^) in iron phosphide crystals (FeP and Fe_2_P). However, the peak centered at binding energy around 712.6 eV is ascribed to a satellite peak of the oxide layer (Fe_2_O_3_) on iron surface.^62,71^ Regarding to XPS results, it can be implied that both phosphate and metal oxide species is probably formed by oxidation of catalyst surface during XPS sample preparation. Also, the formation of unreduced species might represent by an incomplete reduction process.^70,71^ This result is a good agreement with the results of XPS analysis reported by Lui *et al.*,^70^ and Dong *et al.*^71^

### 3.3 Test of catalyst performance in deoxygenation of palm oil

**Fig. 8:**
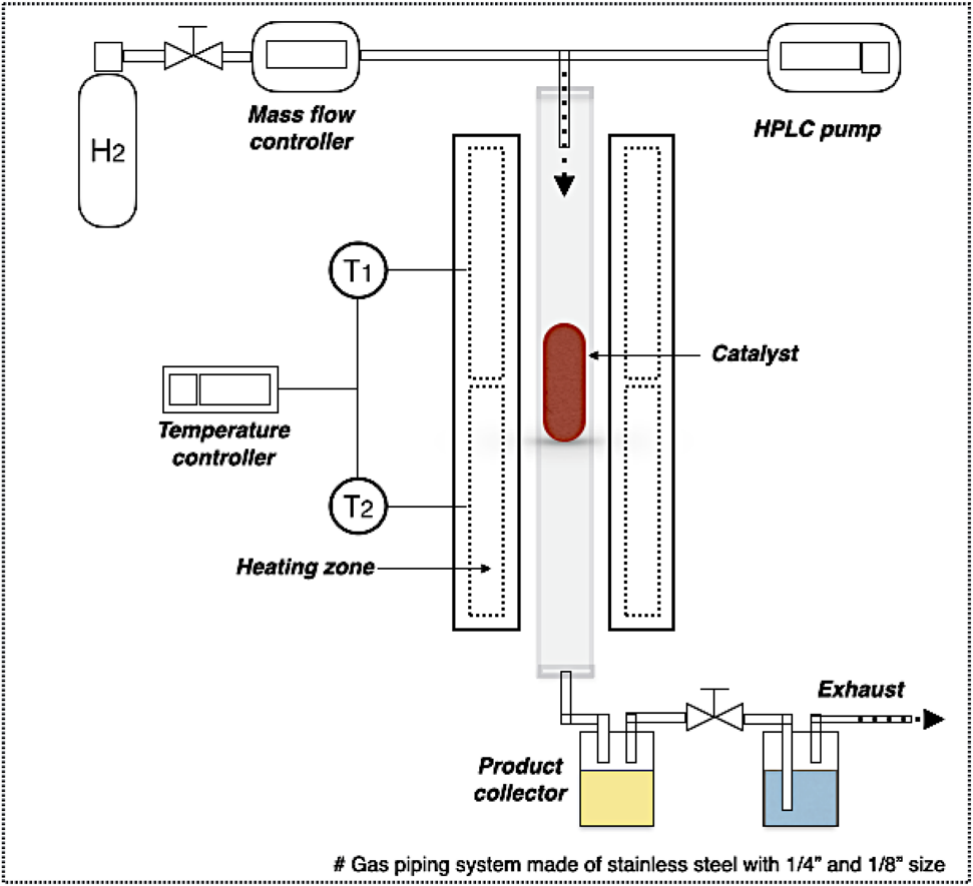
Schematic diagram of deoxygenation unit.

**Fig. 9:**
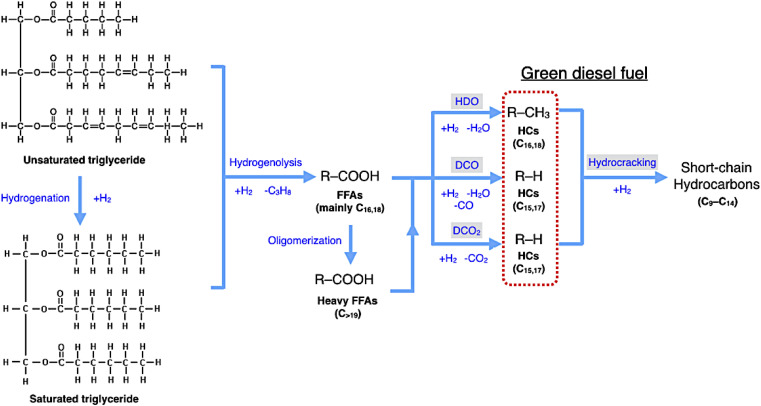
Reaction pathway of palm oil conversion to green diesel.

In DO of palm oil, the relative supported metal phosphide catalysts were evaluated on its catalytic performance for producing of green diesel fuel due to the individual properties of each catalyst, with higher porosity, greater acidity, could be ascribed to the high efficiency alternative catalyst. The palm oil conversion was completely obtained in the trickle bed down-flow reactor at the deoxygenation temperatures of 340–400 °C under the H_2_ pressure of 50 bar using H_2_ flow of 100 mL min^−1^, and the palm oil was fed at the liquid hourly space velocity (LHSV) of 1 h^−1^ by using HPLC pump, as seen in schematic process diagram on Fig. 8. However, the purpose of reaction pathway according to the product distribution during the conversion of palm oil over metal phosphide was clearly illustrated in Fig. 9. Firstly, the unsaturated triglyceride in palm oil were hydrogenated to saturated triglyceride and were then degraded to fatty acid molecules (FFAs) and propane gas as a by-product by hydrogenolysis reaction. Before the deoxygenation stage, the FFAs was probably transformed to larger FFAs molecules *via* oligomerization, which could be assured by the appearance of long-chain hydrocarbon products after deoxygenation.^72^ The green diesel fuel and other hydrocarbons were obtained from the deoxygenation of FFAs through three main reactions (*i.e.* HDO, DCO, DCO_2_), simultaneously. HDO reaction required an excess H_2_ consumption to produce hydrocarbons with the same number of carbon atoms in feedstock by the cleavage of C–O bonding as well as the water as a by-product. For DCO reaction, H_2_ was partially used to form hydrocarbon molecules with CO and water as a by-product, while DCO_2_ reaction does not required H_2_ because the hydrocarbons were produced by C–C bond cleavage with releasing CO_2_ resulted in odd number of carbon atom hydrocarbons.^73^

**Fig. 10:**
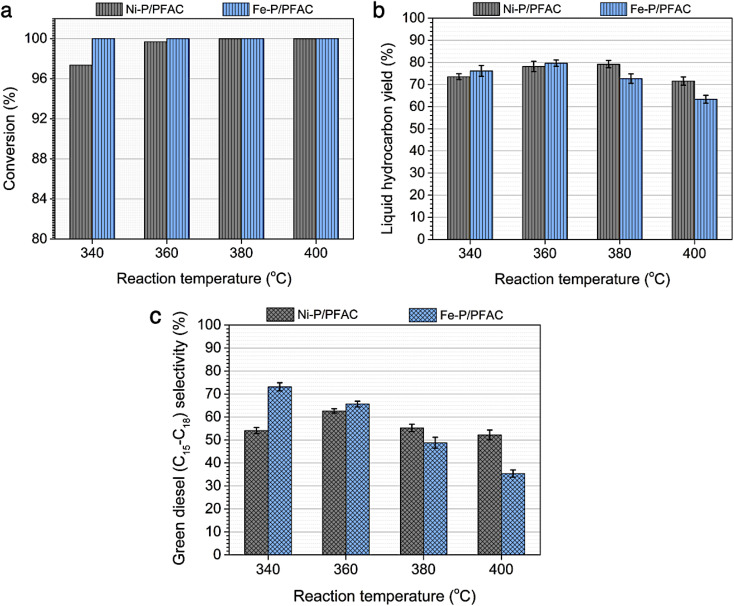
Performance study of Ni–P/PFAC and Fe–P/PFAC catalysts in palm oil deoxygenation for green diesel production at different reaction temperatures (a) palm oil conversion, (b) liquid hydrocarbon yield, and (c) green diesel selectivity.

Additionally, Fig. 10 represented palm oil conversion, yield of liquid hydrocarbon product, and selectivity towards green diesel obtained from different reaction temperatures of 340–400 °C by using Ni–P/PFAC and Fe–P/PFAC catalysts. According to the deoxygenation over Ni–P/PFAC, the reaction at 340 and 360 °C revealed the conversion about 97.36 and 99.69%, while the complete conversions (100%) were obtained from 380 and 400 °C, respectively (Fig. 10a). The incomplete conversion at the reaction temperature below 350 °C in a continuous flow system was consistent with the report from previous study that an incomplete conversion was significantly obtained at low-temperature operation because some intermediates in oxygenated compounds, such as palmitic acid, and stearic acid in the liquid hydrocarbon product could not undergo further to produce hydrocarbons by HDO, DCO, and DCO_2_ reactions.^73^ The evidence implied that supported Ni–P catalyst requires a higher reaction temperature in conversion of vegetable oils for biofuel production.^70,71,73^ In contrast, 100% conversion of palm oil was obtained at the reaction temperatures of 340–400 °C in the deoxygenation over Fe–P/PFAC, as seen in Fig. 10a. This finding implies that iron phosphide has a superior catalyst activity in deoxygenation. However, there are no recent studies reported the catalytic performance of iron phosphide in producing green diesel.

In addition, liquid hydrocarbon yield obtained from the corresponding catalysts are summarized in Fig. 10b. The study of catalytic activity on liquid hydrocarbon from palm oil were compared between Ni–P/PFAC and Fe–P/PFAC catalysts, which obtained at the different testing condition in DO with 100% conversion. The highest liquid hydrocarbon yield of palm oil deoxygenation over Fe–P/PFAC catalyst is approximately 79.65% (obtained at 360 °C). While the total liquid hydrocarbon yield obtained by using Ni–P/PFAC catalyst is about 79.21% (obtained at 380 °C). The liquid hydrocarbon yield was continuously decreased with increasing reaction temperature, due to the decomposition of palm oil and intermediated to gaseous hydrocarbon occurred at high-temperature condition by hydrocracking reaction.^74^ However, the finding results show the beneficial role of Fe–P catalyst at lower-temperature of operating condition compared to Ni–P catalyst. The green diesel (*n*-C_15_–C_18_) selectivity towards carried out from the Ni–P/PFAC and Fe–P/PFAC catalysts at varied deoxygenation temperatures are illustrated in Fig. 10c. According to the deoxygenation over supported Ni–P catalyst, the green diesel selectivity is approximately 52.2–62.6%, while the highest green diesel selectivity (62.6%) was obtained at the reaction temperature of 360 °C. The green diesel fraction was significantly increased by adjusting reaction temperature from 340 to 360 °C. This correlated with the complete palm oil conversion, as seen in Fig. 10a. Additionally, when the reaction temperature was shifted to 380 and 400 °C, the decrease in selectivity of green diesel was obtained. This is due to the hydrocracking of long-chain hydrocarbon significantly occurred at high temperature condition.^74^

However, the superior catalytic activity of supported Fe–P catalyst compared to supported Ni–P catalyst is certainly due to strong acidic characteristic and well dispersion resulted in higher surface of active sites. The highest green diesel selectivity (73.12%) was obtained at lower deoxygenation temperature, compared to Ni–P supported on carbon catalyst. This could be summarized that Fe–P/PFAC catalyst is effective for the deoxygenation at low temperature conditions to maximize the green diesel fraction. In contrast, the selectivity of green diesel was gradually decreased at the reaction temperature greater than 360 °C owning to the transformation of long-chain hydrocarbons to shorter-chain hydrocarbons at high-temperature operation during palm oil deoxygenation over supported Fe–P catalyst. This result shows the potential of support Fe–P catalyst that can be used as a promising alternative for hydrocracking in the production of light hydrocarbon, such as bio-gasoline and bio-jet fuel.^64,74,75^

**Fig. 11:**
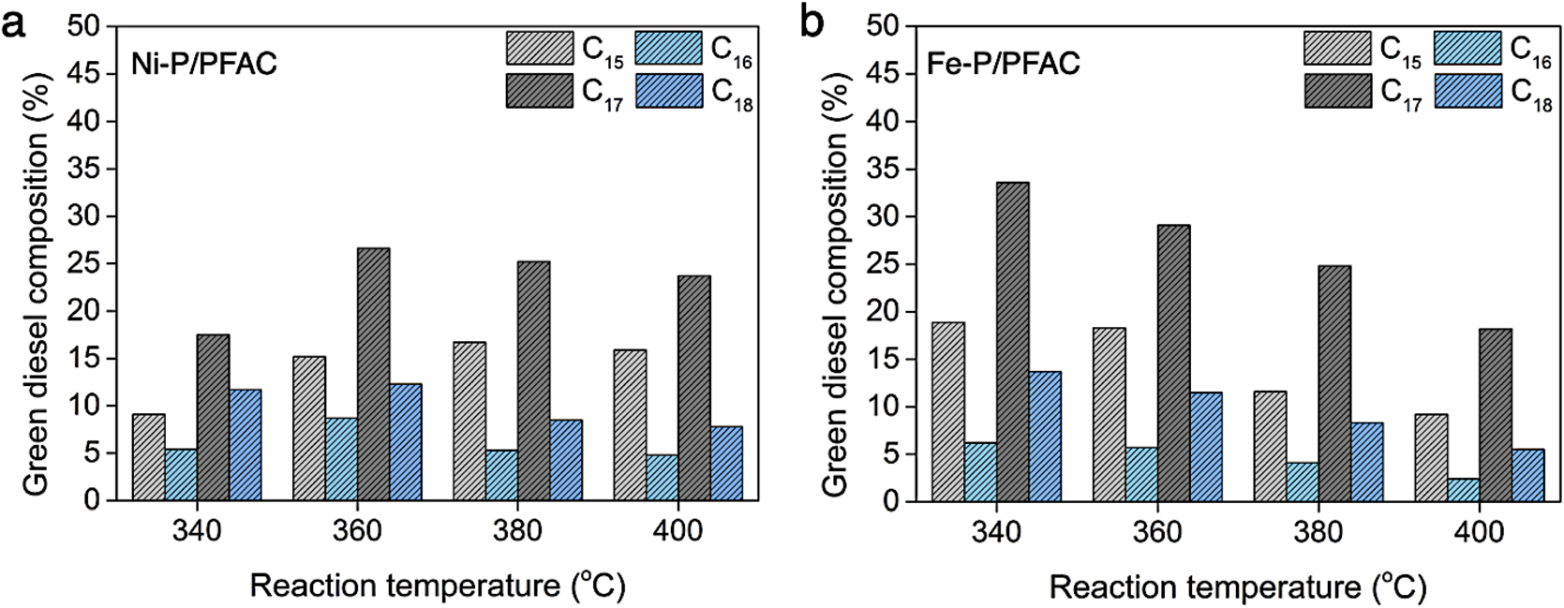
Green diesel composition based on carbon number (a) obtained by using Ni-P/PFAC, and (b) obtained by using Fe-P/PFAC.

Fig. 11 shows the hydrocarbon composition based on carbon number in green diesel fuels. As we known that, palm oil feed stock is mainly composed by unsaturated triglycerides, likes palmitic acid (C16 : 0, 37.8%) and oleic acid (C18 : 1, 45.8%).^74^ Therefore, the major hydrocarbon products should be C_16_ and C_18_ hydrocarbons followed the chemical structure of oil feedstock. However, the product distribution obtained from supported Ni–P and Fe–P catalysts is mainly C_15_ and C_17_ hydrocarbons. The amount of C_15_ and C_17_ hydrocarbons is approximately two times greater than C_16_ and C_18_ hydrocarbons. This evidence suggests that the deoxygenation of palm oil over the corresponding catalysts is transformed though the decarbonylation and decarboxylation as a major reaction. However, the hydrodeoxygenation also existed in the reaction route simultaneously, that is ensured by the formation of C_16_ and C_18_ hydrocarbons as a minor product.^75,76^ In deoxygenation over Ni–P/PFAC catalyst, the major products including C_15_ and C_17_ hydrocarbons was catalyzed by DCO/DCO_2_ due to the presence of Ni_2_P as a dominant active phase. These results are in good correlation with the study from Zhou *et al.*^76^ that reveal the Ni_2_P is favorable for decarbonylation through the scission of C–C bonding resulted in the hydrocarbon products with one less carbon atom (C_*n*−1_) as the main product. Moreover, the Ni_12_P_5_ species exhibited both DCO and HDO simultaneously showing that the hydrocarbon products with similar carbon number of triglycerides in palm oil feedstock (C_*n*_) is also observed. Furthermore, the hydrocarbon product obtained by using supported Fe–P catalyst exhibited the same product distribution (C_15_ and C_17_ hydrocarbons). This result implies that the DCO/DCO_2_ reactions are mainly crucial roles in deoxygenation of palm oil over supported Fe–P catalyst since the high acidity of Fe–P catalyst is important for catalyzing C–O and C–C cleavage to conduct C_*n*−1_ hydrocarbons.^75,77^ Additionally, recent study also ensured that the DCO/DCO_2_ pathways was the favor reaction in deoxygenation over Fe phosphide catalyst.^77^ One remarkable, the observation from XRD analyzes demonstrated that Fe–P/PFAC catalyst particularly consists of the Fe_2_P active phase. This finding can be implied that Fe_2_P species show the similar catalytic behavior with Ni_2_P phase.

Besides, the comparison of current study with several literatures on the green diesel production over different catalysts and experimental conditions are listed in Table 5. In this study, the optimum condition of green diesel production from palm oil by catalytic deoxygenation is at reaction temperature of 340 °C over Fe–P/PFAC catalyst, providing the highest green diesel selectivity. This result could be implied that supported iron phosphide catalysts is a promising for the conversion of triglycerides into renewable diesel fuel due to lower cost than those noble metals, and excellent catalyst activity for production of biofuels.

**Table 5:** Green diesel production from catalytic deoxygenation of current work compares to literatures

Catalyst	Reactant	Condition	GD selectivity (%)	Ref.
30 wt% Ni–Co/MWCNT	Jatropha curcas oil	350 °C, *T* = 1 h, 5% loading 10 mbar (Batch reactor)	64	9
20 wt% NiO–ZnO	By-product (palm oil refining process)	350 °C, *T* = 2 h, 5% loading, N_2_ atmosphere (Batch reactor)	86	10
20 wt% MoP/SBA-15	Methyl ester	290 °C, H_2_ = 30 bar, WHSV = 20.1 h^−1^ (fixed bed, micro reactor)	<90%	69
5 wt% Pd–Fe/Al_2_O_3_	Palm oil	400 °C, *T* = 2 h, H_2_ = 60 bar, 1% loading (High pressure batch reactor)	62.2	75
5 wt% Ni–Co/SBA-15	Palm oil	350 °C, *T* = 2 h, 10% loading (without H_2_, Batch reactor)	78	55
Fe–P/activated biochar (10 wt% of FeP)	Palm oil	340 °C, H_2_ = 50 bar, LHSV = 1 h^−1^ of palm oil feed (continuous-flow reactor)	73.1	This work

## 4. Conclusions

Palm fiber waste derived activated biochar (PFAC) was successfully prepared by KOH activation process. The PFAC is efficiently applied as a catalyst support due to its special characteristics, such as high specific surface area and good mechanical strength. The synthesis of supported Ni–P and Fe–P catalysts was done by the wetness impregnation technique. The application of activated biochar as a support material could enhance metal phosphide dispersion resulted in higher catalyst activity. Green diesel fuel was completely produced over PFAC supported Ni–P and Fe–P catalysts in palm oil deoxygenation. This study shows that palm fiber waste derived activated biochar is suitable to be used as a support material for Ni–P and Fe–P catalysts in the catalytic deoxygenation of palm oil for producing green diesel fuel. The highest selectivity towards green diesel (73.1%) was obtained by using supported Fe–P catalysts at the deoxygenation temperature of 340 °C with a complete of palm oil conversion. The second highest green diesel selectivity (62.6%) was obtained by using supported Ni–P catalysts obtained at the reaction temperature of 360 °C. Interestingly, the liquid hydrocarbon product is mainly composed of C_15_ and C_17_ hydrocarbons indicating that high proportion Ni_2_P and Fe_2_P pure phases on supported metal phosphide catalysts are favored to DCO/DCO_2_ reactions. One remarkable result is the hydrocracking of long-chain hydrocarbon could be occurred at the condition of high-reaction temperature. Although, the deoxygenation performance of PFAC supported metal phosphide catalysts is not superior when compared to those supported noble metal catalysts (*i.e.* Pt-, Pd-, Rh-based catalysts), the synthesized catalyst exhibited better green diesel selectivity than 5 wt% Pd–Fe/Al_2_O_3_ (seen in Table 5). Hence, the further improvement of deoxygenation activity for biofuel production is still challenge on the catalyst selection, synthesis condition, and performance testing. From findings, this study provides the beneficial knowledges, which full fill the information gaps reference to the potential utilization of waste biomass derived porous carbon applied as a support material for DO catalysts. Nonetheless, the development of desired product contributions in the yield and selectivity of green diesel could be achieved by optimizing and find-tuning experimental variables, such as types of catalysts, loading content, and reaction conditions in palm oil deoxygenation for further study.

## Authors contributions

Napat Kaewtrakulchai: conceptualization, formal analysis, investigation, data curation, writing–original draft Masayoshi Fuji: conceptualization Apiluck Eiad-ua: writing–review and editing, conceptualization, supervision funding. This research received no external funding

## Conflicts of interest

The authors declare that they have no known competing financial interests or personal relationships that could have appeared to influence the work reported in this publication.

## Supplementary Material
